# Comparative transcriptomic analysis of chicken immune organs affected by Marek’s disease virus infection at latency phases

**DOI:** 10.3389/fphys.2025.1520826

**Published:** 2025-04-02

**Authors:** Yi Ding, John Dunn, Huanmin Zhang, Keji Zhao, Jiuzhou Song

**Affiliations:** ^1^ Allen Institute for Brian Science, Seattle, WA, United States; ^2^ U.S. Department of Agriculture, U.S. National Poultry Research Center, Agricultural Research Service, Athens, GA, United States; ^3^ Laboratory of Epigenome Biology, Systems Biology Center, National Heart, Lung and Blood Institute, NIH, Bethesda, MD, United States; ^4^ Department of Animal and Avian Sciences, University of Maryland, College Park, MD, United States

**Keywords:** Marek’s disease, Marek’s disease virus, poultry health, immune organs, gene exression

## Abstract

Over the past decades, MDV has dramatically evolved towards more virulent strains and remains a persistent threat to the world’s poultry industry. We performed genome-wide gene expression analysis in the spleen, thymus, and bursa tissues from MD-resistant line and susceptible line to explore the mechanism of MD resistance and susceptibility. We identified genes and pathways associated with the transcriptional response to MDV infection using the robust RNA sequencing approach. The transcriptome analysis revealed a tissue-specific expression pattern among immune organs when confronting MDV. At pathway and network levels, MDV infections influenced cytokine-cytokine receptor interaction and cellular development in resistant and susceptible chicken lines. Meanwhile, we also observed different genetic responses between the two chicken lines: some pathways like herpes simplex infection and influenza A were found in MD resistant line spleen tissues, whereas metabolic-related pathways and DNA replication could only be observed in MD susceptible line chickens. In summary, our research renders new perceptions of the MD progression mechanism and beckons further gene function studies into MD resistance.

## Introduction

Marek’s disease (MD) is a highly contagious and neuropathic disease in chickens caused by Marek’s disease virus (MDV), which is a cell-associated alphaherpesvirus that transforms T lymphocytes and triggers mononuclear tissue infiltration in tissues such as peripheral nerves, muscle, visceral organs, and skin (Bacon et al., 2000; Biggs, 2001; Witter et al., 2005). MDV infection involves the early cytolytic stage, latency phase, and late cytolytic phase; at each stage of infection, the virus targets immune components of the host system (Tian et al., 2012). MDV confronts primary target cells (B lymphocytes and activated CD4^+^ T lymphocytes) and reaches replication peak from three to 7 days post-infection (Shek et al., 1983; Baigent and Davison, 1999; Baigent et al., 1998). 7–8 days after infection, MDV progression switches from cytolytic phase to latency without producing infectious progeny in activated CD4^+^ T cells and infected B cells. Reactivation from latency to late cytolytic infection occurs around 2–3 weeks post-infection in susceptible chickens (Osterrieder et al., 2006). The reactivation period (late cytolytic phase) is usually companioned with tumor formation and other acute disease symptoms.

High-throughput gene expression analysis has been widely used to understand host-virus interactions. Before the RNA-Seq method, microarrays were helpful to detect host gene expression in chicken embryo fibroblasts (Morgan et al., 2001) and spleen tissue (Sarson et al., 2006; Heidari et al., 2010; Kano et al., 2009a) using MD-resistant and MD-susceptible chicken models (Sarson et al., 2008; Yu et al., 2011; Smith et al., 2011). *CD8A*, *IL8*, *USP1*, and *CTLA4* genes were considered as significant genes associated with MD-resistance or–susceptibility by testing temporal transcriptome changes using three representative chicken lines (Yu et al., 2011). By taking advantage of next-generation sequencing techniques, researchers have characterized differential expression of genes in the spleen of broiler and layer chickens, finding that TLR receptor and JAK/STAT signaling pathways were enriched following MDV infection (Perumbakkam et al., 2013).

Well-defined chicken models involve two highly inbred chicken lines 6_3_ and 7_2_, sub-lines of lines 6 and 7, which have been bred since 1939 and served as unique resources to explore the mechanistic difference towards MD response (Bacon et al., 2002). Availability of chicken genome sequence (Hillier et al., 2004) and next-generation sequencing techniques have altered our capability to identify more critical factors for MD resistance using these two chicken lines. Here we conducted genome-wide profiling of spleen, thymus, and bursa transcriptomes in MD-resistant line 6_3_ and MD-susceptible line 7_2_ using mRNA sequencing (RNA-Seq). Age-matched controls aiming at testing innate distinction among inbred chicken lines and those induced by MDV infection were implemented in our experimental design. Also, comparisons among immune organs would further uncover gene candidates related to different MD response.

## Materials and methods

### Animals and experimental design

Two inbred lines of White Leghorn (line 6_3_ and line 7_2_) were hatched, raised and maintained in USDA-ARS Avian Disease and Oncology Laboratory (Michigan, United States). Chickens from each line were separated into infected and non-infected groups respectively. Chickens from the infected group were injected intra-abdominally with a partially attenuated virulent plus strain of MDV (648A passage 40) at 5 days after hatching with a viral dosage of 500 plaque-forming units (PFU) (Chang et al., 2010). At 21 days post-infection, five chickens from each treatment and control group were sacrificed following standard animal ethics and usage guidelines. Samples from spleen, thymus, and bursa organs were gathered, frozen and stored at −20°C until RNA extraction.

### RNA preparation and sequencing

Two replicates of spleen, bursa, and thymus samples were randomly selected from infected and control groups from MD-resistant line 6_3_ and MD-susceptible line 7_2_ chickens. Approximately 30∼50 mg of the spleen, thymus, and bursa tissues were homogenized in TRizol Reagent (Qiagen, United States), and total RNA in infected and control groups was extracted by using RNeasy Mini Kit (Qiagen, United States). The mRNA isolation was performed using Oligotex mRNA Mini Kit (Qiagen United States) according to the manufacturer’s instruction. Biological replicates were applied for further RNA-Seq library construction and analysis. About 300 ng of mRNA was used to synthesize the first and second strands of cDNA by SuperScriptTM III Reverse Transcriptase (Invitrogen, United States) and Second Strand cDNA Synthesis Kit (NEB, United States). After sonication, the dscDNA fragment ends were repaired by T4 and Klenow DNA polymerase and underwent library construction procedures following Illumina. Each library was identified by adding a 6-bp adaptor and sequenced at 50-bp read by an Illumina HiSeq 2,500 Sequencer.

### Differential expression and pathway analysis

Raw sequencing data were checked for quality considering read counts, overall read quality, and read distribution, etc. The alignment to the reference genome (ICGSC Gallus_gallus-4.0/galGal4) downloaded from the UCSC Genome Brower (http://genome.ucsc.edu/) was performed by using an ultrafast memory-efficient short read aligner Bowtie (Langmead and Salzberg, 2012). First 15 bps from the original raw 50-bp reads were trimmed to control the mapping quality. The counting matrix was generated by the summarizeOverlaps function in R. R package edgeR (Robinson et al., 2010) and corresponding complementary functions GLM were executed to analyze count reads data and perform comparative analysis. The threshold of differentially expressed genes was set as 0.1 FDR. David (Huang et al., 2007) and Ingenuity Pathways Analysis (IPA) (Dessì et al., 2011) were utilized to analyze the biological process, molecular functions and pathways enriched for those differentially expressed genes.

### Principal component analysis and Venn diagram construction

After alignment of sequencing reads and the generation of counting matrix by summarizeOverlaps function in R, an integrated, normalized data matrix for the spleen, bursa, and thymus was created using Bioconductor’s DESeq (Anders and Huber, 2010) package. Principal components facilitate dimensionality reduction and noise filter, which identifies directions of maximum variance in the original data. The function *prcomp* in the R statistic package was used to perform PCA on the integrated, normalized data matrix. The top PCs were visualized using the factoextra package in R. The Venn diagram was constructed using Venn diagram function in R.

### Quantitative real-time RT-PCR

Several significant genes based on RNA-Seq analysis were validated by real-time PCR using the synthesized dscDNA described previously (Yu et al., 2011). Real-time reactions were conducted with an iQ SYBR Green PCR Kit (Bio-rad, United States) according to manufacturer’s instructions. Primers were designed using Primer3 (http://fokker.wi.mit.edu/primer3/input.htm). The melting temperature was 60, and the length of amplicons was between 50 and 200bp. Ct values were calculated based on normalization of the housekeeping gene GAPDH, and three technical replicates were performed.

## Results

### Gene expression profiles of immune organs in MDV challenge experiments

**FIGURE 1:**
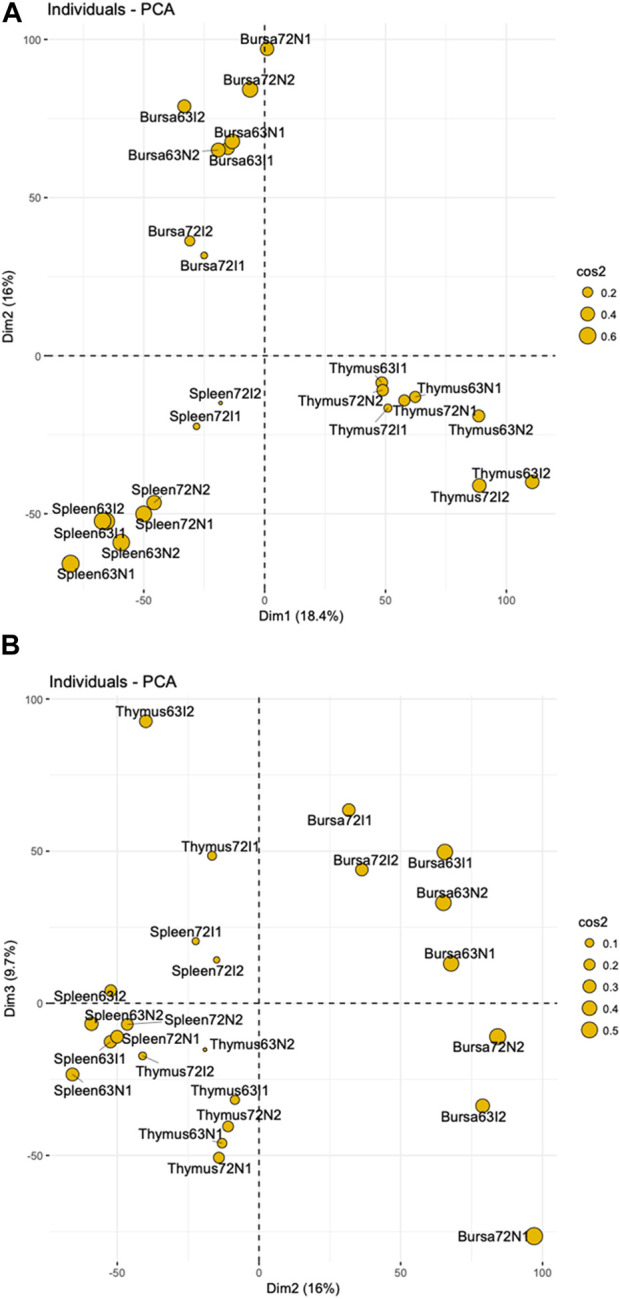
PCA Plots **(A)** a two-dimensional PCA plot showing principal component 1 (Dim1) and principal component (Dim2) of 24 individuals indicate tissue-specific gene expression patterns with a slight similarity between chicken spleen and thymus organs. **(B)** a two-dimensional PCA plot showing principal component 2 (Dim2) and principal component 3 (Dim3) across spleen, thymus, and bursa tissue. In both figures, the percentages represent the proportion of the total variance in the data that is captured by each principal component. Cos2 values indicate quality of the representation for individuals on the principal components. A higher cos2 represents a better representation of the individual on the principal component.

To discover genes involved in MDV response, we performed transcriptome analysis on the spleen, thymus, and bursa of Fabricius of two chicken lines at 21 days post-MDV infection. An integrated gene expression dataset of three immune organs was created as described in Materials and Methods—the data matrix contained normalized gene expression measurements of 24 samples for 17,108 genes (Supplementary Table S1). To explore whether the samples would form distinct groups based on their gene expression profiles, we used principal component analysis (PCA) on the data matrix, and the results were visualized by scatter plots (Figures 1A, B). The preliminary PCA plot indicated the chicken samples tended to cluster by organs regardless of MDV infection when we examined the first two principal components, indicating tissue-specific gene expression patterns after MDV infection. Also, the similarity of gene expression patterns between the spleen and thymus was higher compared to other combinations when we plotted PC2 and PC3 as shown in Figure 1B.

### Genome-wide profiling of the spleen transcriptome at the late cytolytic phase

For the spleen tissue, more than 211 million sequence reads were generated and mapped to the chicken genome (galGal4). Read counts associated with annotated Ensembl genes (Flicek et al., 2014) the statistical package edgeR profiled was calculated, and differential gene expression (Robinson et al., 2010). Comparisons were carried out between infected and control birds within each line, and birds underwent non-infection status from two lines to indicate disease response and baseline transcription differences.

**FIGURE 2:**
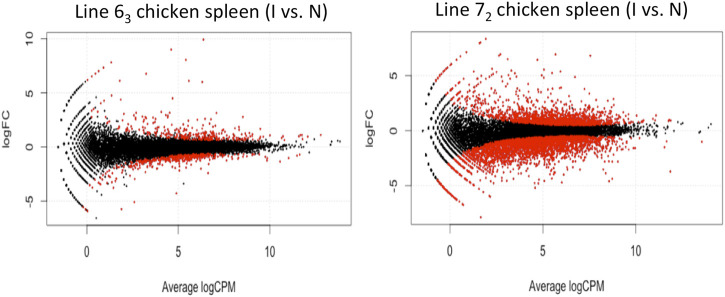
Differentially Expressed Genes in Chicken Spleen Tissues in the volcano plot In chicken spleen tissues, the analysis detected 817 differentially expressed genes between MDV-infected and non-infected L63 chickens (left panel) and 4,584 genes in L7_2_ chickens (right panel) with FDR < 0.1. Each red dot represents the significant individual differentially expressed gene.

The behavior discrepancy between the two chicken lines (MD-resistant and MD-susceptible) is remarkable when challenged by MDV infection. We witnessed a significantly larger number of differentially expressed (DE) genes in the comparison between MDV-infected and non-infected line 7_2_ chickens (susceptible chicken line) than that between MD-resistant chickens. In general, the analysis detected 817 differentially expressed (DE) genes between infected and control MD-resistant line 6_3_ chickens, and 4584 DE genes in MD-susceptible L7_2_ chickens using a threshold of FDR<0.1 (Figure 2). Two chicken lines share 522 DE genes regardless of the changing direction. The direction of changes in these two lines is similar: most of the DE transcripts in the resistant line 6_3_ (563 out of 817, 68.91%) and the susceptible line 7_2_ (2,959 out of 4,584, 64.55%) were downregulated. In those shared 522 DE genes, 136 genes were upregulated both in MD-resistant line 6_3_ and MD-susceptible line 7_2_ infected birds, and 365 genes were downregulated, while the remaining 21 genes displayed opposite alteration trends in resistant and susceptible chicken lines.

When comparing the innate difference between two chicken lines, most DE genes (801 out of 1,118, 71.64%) exhibited higher expression levels in MD-resistant line 6_3_. Almost half (49%) of these genes were also differentially expressed in susceptible line 7_2_ in response to MDV infection (84.49% downregulated). Those distinctions between the two inbred chicken lines might reflect the baseline transcription that could be the possible contribution of varying MD-resistant phenotypes.

### Thymus and bursa transcriptome profiling at late cytolytic phase

**FIGURE 3:**
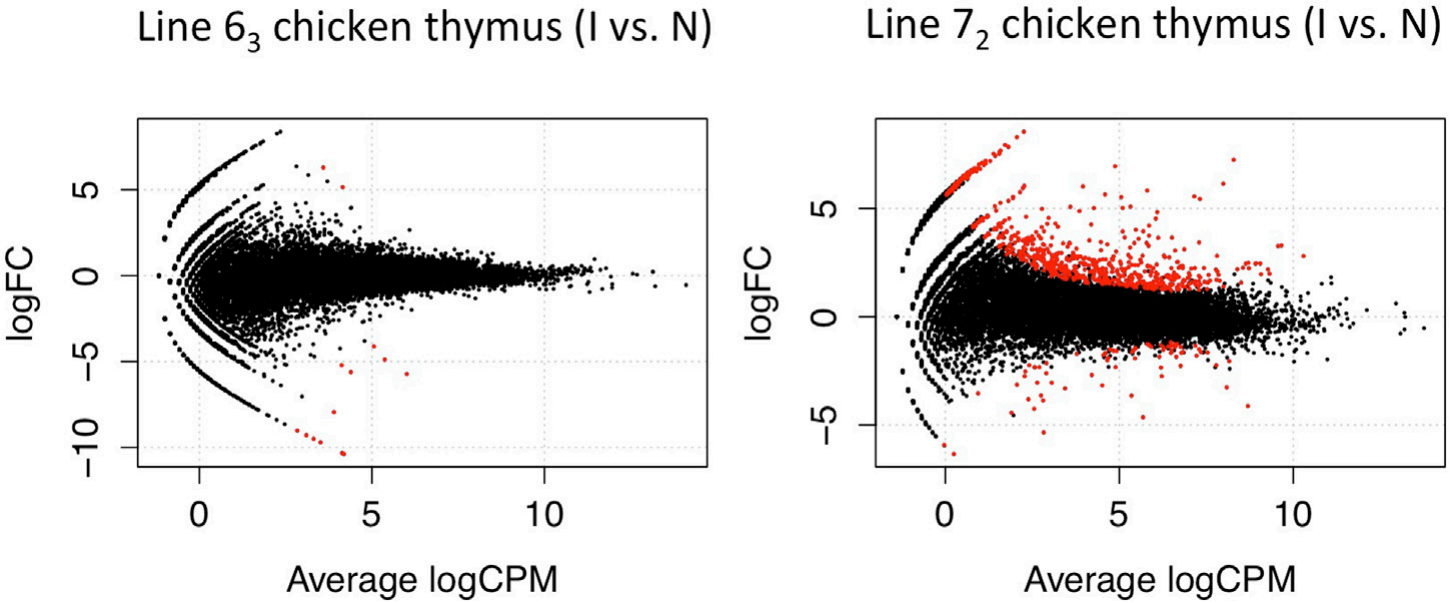
Differentially Expressed Genes in Thymus Tissue. The volcano plot shows the discovered 734 significant differentials expressed genes in susceptible line 7_2_ chickens while 14 DE genes in resistant line 6_3_ birds using a threshold of FDR < 0.1 in the thymus tissue (red dots).

**FIGURE 4:**
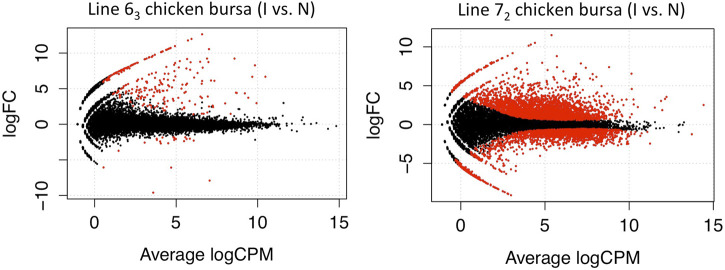
Differentially Expressed Genes in Bursa Tissue In the bursa organ, 259 and 5387 DE genes were detected in line 6_3_ and line 7_2_ chickens respectively with FDR < 0.1.

Following a similar experimental design and analysis methodology, we examined the genome-wide transcription levels in the thymus and bursa organs at the late cytolytic phase for the above chicken lines. Similarly, considerably larger DE genes were observed in MD-susceptible line 7_2_ birds for both tissues. In thymus tissues, we discovered 734 DE genes in susceptible chicken lines while only 14 DE genes in resistant chicken lines using a threshold of FDR < 0.1 (Figure 3). In the bursa organ, 259 and 5387 DE genes were detected in resistant and susceptible chickens, respectively with FDR < 0.1 (Figure 4). Interestingly, most DE genes were upregulated in response to MDV infection in the bursa and thymus tissues except for MD-resistant line 6_3_ chicken thymus.

Comparisons between control birds in thymus and bursa organs revealed the intrinsic difference between chicken lines. Approximately 57% (266 out of 465) DE genes showed higher expression levels in the thymus of resistant line 6_3_ chickens and 54.74% (1,542 out of 2,817) in the bursa of resistant line 6_3_ chickens. In summary, by conducting two sets of comparisons in different immune organs, we were able to uncover the innate and MDV-induced variation in two inbred chicken lines. A slightly larger proportion of genes exhibited higher baseline expression levels in the resistant line. However, more significant DE genes were detected in the susceptible chickens than in the resistant chickens at the late stage of infection, probably due to transcriptional activation during the late cytolytic phase in MDV-susceptible chickens.

**FIGURE 5:**
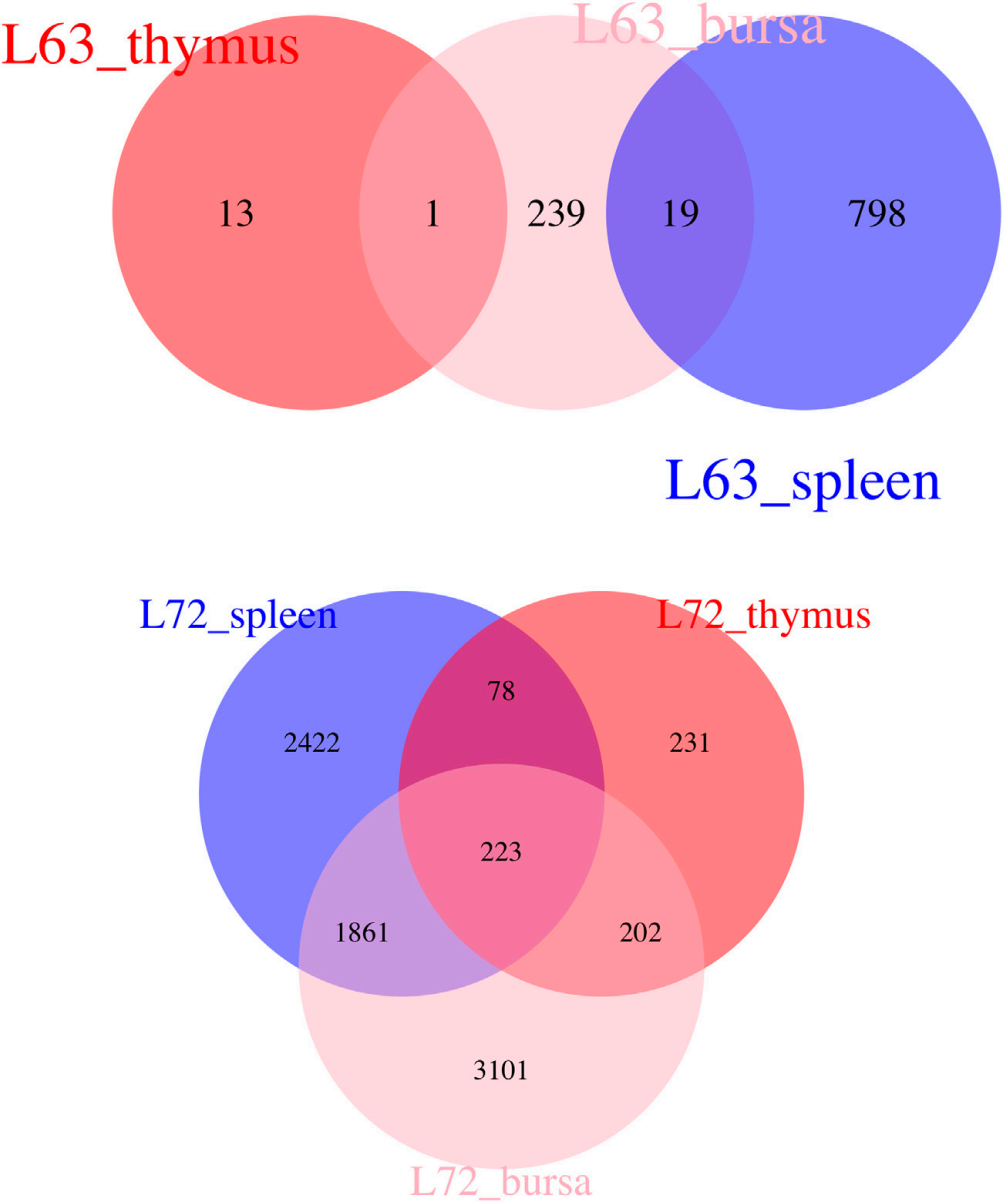
Venn Diagram Indicating Overlap Genes The Venn diagram indicates the overlap differentially expressed genes among different organs after Marek’s disease virus infection.

Witnessing the distinct features of these chicken lines, we attempted to characterize genes associated with MD resistance and–susceptibility to pair-wise comparisons. We made the following hypothesis: genes differentially expressed after MDV infection in all three immune organs are likely a reflection of the typical host response and an immune network to viral infection; genes that are differentially expressed merely in one immune tissue could be part of an organ-specific host response and function to viral infection. According to this rationale, we identified common responses and organ-specific functional genes after MDV challenges (Figure 5). Most DE genes were only present in one tissue, indicating the tissue-specific expression patterns of MDV infection. Based on the KEGG pathway, the significant pathway for those shared DE genes involved the cytokine-cytokine receptor interaction.

### Pathway analysis to reveal networks and biofunctions involved in MDV infection

Previous studies have identified some well-known immune factors in the DE gene list. These include chemokine receptor 1 (Xcr1), chemokine receptor 2 (CCR2), interleukin 1 receptor, type II (IL1R2), type I (IFNA) interferon, and type II (IFNG) interferon. Additionally, several other innate immune function genes were influenced, such as FYN oncogene related to SRC, FGR, YES (FYN), and CD247 molecule (cd247), which are involved in natural killer cell-mediated cytotoxicity. Interestingly, some profoundly affected genes have no known function in innate immune responses, including collagen type II alpha 1 (COL2A1) and growth factor receptor-bound protein 2 (GRB2).

To identify enriched biological function and networks associated with the differentially expressed genes after MDV infection, we utilized the Ingenuity Pathway Analysis (IPA) and DAVID Bioinformatics Resources to analyze the gene sets obtained by comparing to age-matched uninfected controls. Top networks (from IPA) influenced by MDV infection in three organs are shown in Table 1. From these networks, we noticed that the virus has a broad impact on host gene expression in three immune organs of two chicken lines. Also, if we considered those genes associated with diseases and bio-functions, we discovered that many genes were influenced by MDV infection, including those involved in metabolism, tissue development, and immune-related disorders. Those genes indicated broad similarities in the host response to MDV infection. Nevertheless, some unique networks, such as digestive system development and function, gastrointestinal disease, and hepatic system development and function, were only shown in the spleen of MD-susceptible line 7_2_ chickens after MDV infection (Table 1). To gain more insight into the bio-function associated with MDV infection, we utilized the IPA program to analyze disease and bio-function of those differentially expressed genes (Table 2). As might be expected, cancer and immunological illness were observed.

**TABLE 1:** Enriched networks in organs of chicken lines after MDV infection.

Lines	Organ	Enriched network functions
L6_3_	Spleen	Cellular Development, Embryonic Development, Nervous System Development and Function
		Cancer, Respiratory Disease, Developmental Disorder
		Cell Death and Survival, Cellular Development, Cellular Function and Maintenance
		Developmental Disorder, Hereditary Disorder, Immunological Disease
		Cellular Function and Maintenance, Connective Tissue Development and Function
	Bursa	Cell-To-Cell Signaling and Interaction, Hematological System Development and Function, Immune Cell Trafficking
		Cardiac Hypertrophy, Cardiovascular Disease, Developmental Disorder
		Cellular Development, Embryonic Development, Organismal Development
		Cell-mediated Immune Response, Cellular Development, Cellular Function and Maintenance
	Thymus	Cardiac Arrythmia, Cardiovascular Disease, Organismal Injury and Abnormalities
		Cell Death and Survival, Cell Morphology, Cellular Compromise
L7_2_	Spleen	Cell-To-Cell Signaling and Interaction, Cell Signaling, Cell Morphology
		Post-Translational Modification, Dermatological Diseases and Conditions, Developmental Disorder
		Digestive System Development and Function, Gastrointestinal Disease, Hepatic System Development and Function
		Hematological Disease, Metabolic Disease, Protein Synthesis
		Embryonic Development, Organismal Development, Cellular Movement
	Bursa	Cellular Development, Cellular Growth and Proliferation, Hematological System Development and Function
		Cell-mediated Immune Response, Cellular Function and Maintenance
		Carbohydrate Metabolism, Cell Death and Survival, Cellular Movement
		DNA Replication, Recombination, and Repair, Developmental Disorder
	Thymus	Infectious Disease, Connective Tissue Disorders, Dental Disease
		Metabolic Disease, Lipid Metabolism, Small Molecule Biochemistry
		Humoral Immune Response, Protein Synthesis, Inflammatory
		Response Cell-To-Cell Signaling and Interaction, Cell Signaling, Molecular Transport
		Cell Cycle, Cellular Development, Cellular Growth and Proliferation

**TABLE 2:** Top disease and bio-functions in chicken organs after MDV infection.

Lines	Organ	Diseases and disorders	Physiological system development and function
L6_3_	Spleen	Metabolic Disease, Inflammatory Response, Immunological Disease, Cancer, Organismal Injury and Abnormalities	Cardiovascular System Development and Function, Hepatic System Development and Function, Tissue Development, Connective Tissue Development and Function, Embryonic Development
	Bursa	Cancer, Organismal Injury and Abnormalities, Inflammatory Response	Hematological System Development and Function, Cell-mediated Immune Response, Hematopoiesis, Lymphoid Tissue Structure and Development, Immune Cell Trafficking
	Thymus	Cardiovascular Disease, Organismal Injury and Abnormalities, Connective Tissue Disorders, Developmental Disorder, hereditary Disorder	Cardiovascular System Development and Function, Embryonic Development, Organ Morphology, Organismal Development, Skeletal and Muscular System Development and Function
L7_2_	Spleen	Cancer, Gastrointestinal Disease, Organismal Injury and Abnormalities, Reproductive System Disease, Hepatic System Disease	Cardiovascular System Development and Function, Organismal Survival, Organismal Development, Tissue Morphology, Embryonic Development
	Bursa	Cancer, Organismal Injury and Abnormalities, Inflammatory Response, Hematological Response, Immunological Disease	Hematological System Development and Function, Tissue Morphology, Hematopoiesis, Cell-mediated Immune Response, Lymphoid Tissue Structure and Development
	Thymus	Cancer, Inflammatory Response, Infectious Disease, Cardiovascular Disease, Gastrointestinal Disease	Tissue Morphology, Organismal Development, Hematological System Development and Function, Immune Cell Trafficking, Humoral Immune Response

**FIGURE 6:**
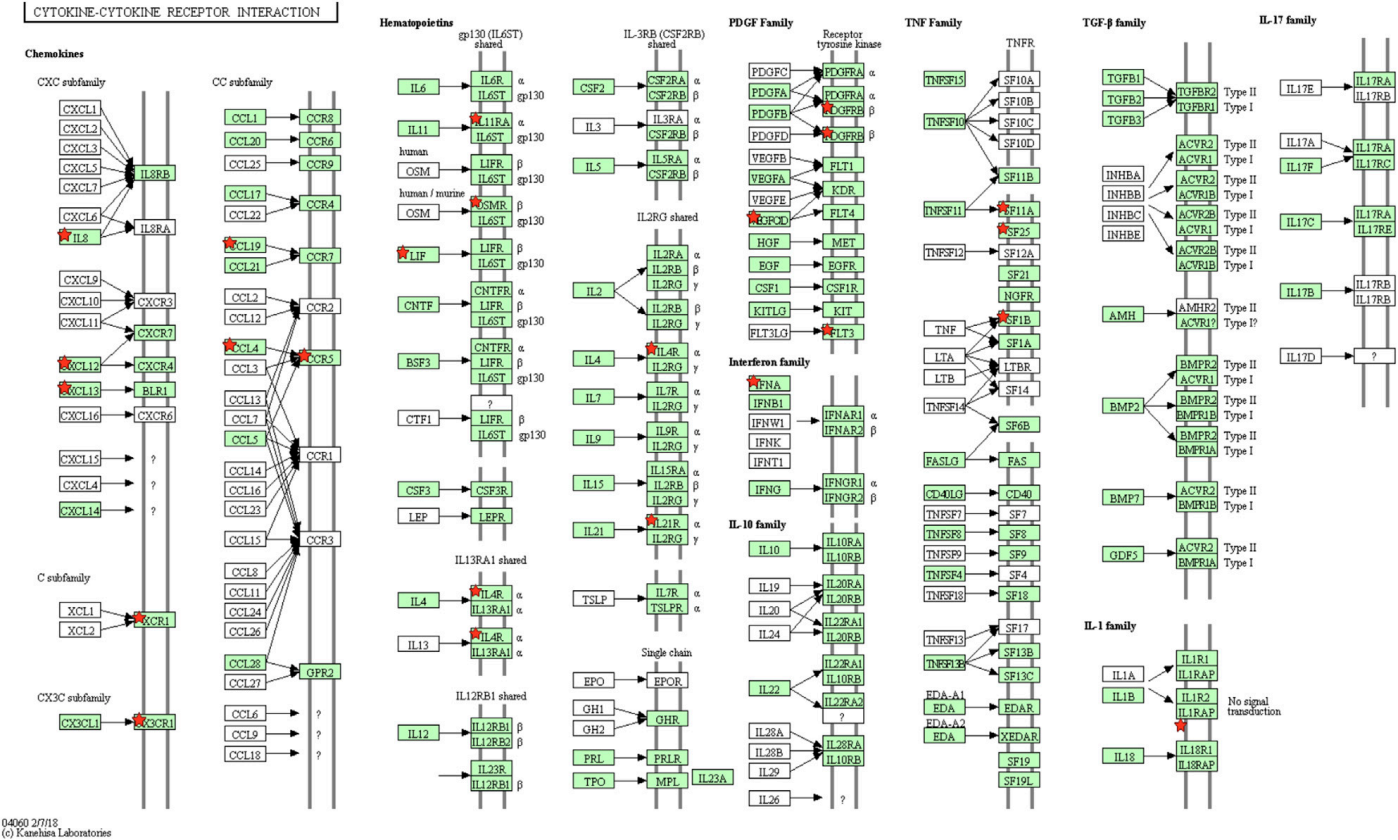
Cytokine-cytokine Receptor Interaction Pathway. One example of the enriched pathway induced by MDV infection in line 6_3_ chickens. Several identified genes from this pathway display significant differential expression patterns, as indicated by red stars in the figure.

Complementary to the discoveries from IPA, DAVID displays biological pathways that were altered (compared to a relevant control) during the host response to MDV infection. Based on KEGG pathways (Kanehisa and Goto, 2000), immune-related pathways, such as cytokine-cytokine receptor interaction (Figure 6), phagosome, herpes simplex infection, and lysosome, were involved in MDV infection response. Also interestingly we could notice the changes in focal adhesion and tight junction pathways, and this might relate to the viral progression in host bodies since MDV requires cell-to-cell contact for dispersal (Smith et al., 2011). The virus might promote such communication by altering the expression of related tight junction formation genes.

### The potential native dissimilarity between lines makes the resistance different

The underlying mechanism for the different responses to MDV infection between two lines might be complex since MD is a complex disease. One possible hypothesis is that before MDV infection, MD-resistant line 6_3_ chickens could promote better immune system development and higher expression of innate immune-related genes compared to MD-susceptible line 7_2_ chickens, while after MDV infection, MD-resistant line 6_3_ chickens mount more robust induced immune responses by regulating adaptive immunity gene activities. As demonstrated previously, baseline transcription differences were discovered between two lines (1118 DE genes in the spleen, 465 DE genes in the thymus, and 2817 DE genes in the bursa) in tissues from the control (uninfected) birds. Among those genes, examples like Apoa4, C3, IL1RL1, LOC395914, Rag2, Smad5, CATHL2, TLR4, TNFRSF1A, Cxcl14, and IL1B are involved in immune system development and innate immune response.

Gene expression disparity was also witnessed between these two lines after MDV infection (3852 DE genes in the spleen, 222 DE genes in the thymus, and 1439 DE genes in the bursa). We could notice changes of adaptive immunity genes including IL18, il10, IRF7, TAP2, SWAP70, Fas, CD28, TNFSF13B, TNFRSF13C, B-MA1, CD40LG, ada, HSPD1, PRKCD, and IFNG. For example, if we focused on the DE genes that only existed in the spleen tissue of MD-resistant line 6_3_ but were not seen in the spleen tissue of MD-susceptible line 7_2_ chickens after MDV infection, we would detect two exciting pathways: toll-like receptor signaling pathway and herpes simplex infection pathway. Therefore, regardless of MDV infection, the inborn discrepancy between the two lines might contribute to the MD resistance mechanism.

### Experimental validation of differentially expressed genes

**FIGURE 7:**
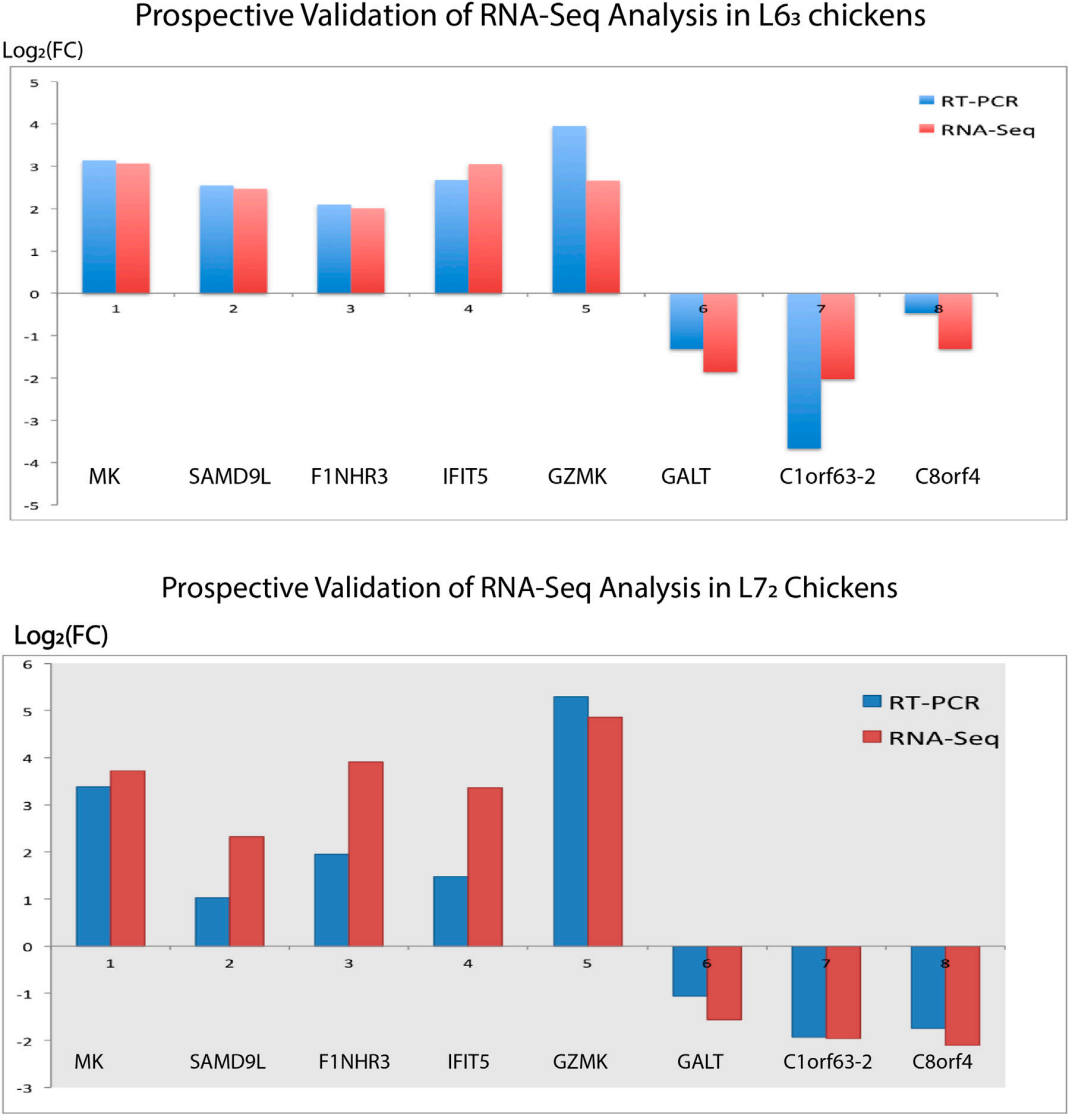
Real-time PCR Validation. Eight genes (five upregulated genes and three downregulated genes) identified in the spleen tissues of L6_3_ and L7_2_ chickens were selected for validation, respectively. The gene expression trend is confirmed if the expression fold change in qPCR analysis is concordant as measured by RNA-seq analysis.

We randomly selected several DE genes to confirm the RNA-Seq reliability and differential expression among contrasting treatment groups. We analyzed the sample using qPCR with dscDNA as the template from the sequencing samples. A reference gene with stable expression is necessary to avoid distortions in qPCR, so GAPDH was used as an internal reference (Sarson et al., 2008). Notably, eight genes were examined in the spleen organ in both MD-resistant L6_3_ and MD-susceptible L7_2_ chickens in response to MDV infection (Figure 7). These eight genes manifested a concordant change direction compared to the RNA-Seq analysis estimate, and the gene expression difference was verified. In general, the validation outcomes from the qPCR assay provide us with further confidence in our analysis.

## Discussion

With extensive studies, cytokine production in response to MDV infection and their potential immune functions against MD have been described including IL4 (Heidari et al., 2008), IL6 (Kaiser et al., 2003; Abdul-Careem et al., 2007), IL10 (Lian et al., 2012), IL18 (Smith et al., 2011; Kaiser et al., 2003; Abdul-Careem et al., 2007), and IFN-^©^ (Xing and Schat, 2000; Kano et al., 2009b). For instance, several studies together indicated the lacking association of IFN-^©^ expression with resistance against MD (Abdul-Careem et al., 2007). Meanwhile, increased IL6 and IL18 expression levels were observed in splenocytes of susceptible chickens. However, those cytokines alone do not adequately manifest interactions among vital immunological mediators and their receptors.

We noticed several interesting DE genes including a set of chemokine receptors and interleukin receptors. For example, chemokine receptors XCR1, CCR2, and CX3CR1 were upregulated in the resistant line’s spleen tissue at the late cytolytic phase, and interleukin receptors IL1R2, IL21R, IL4R, and IL11RA underwent dramatic expression changes. The XCL1-XCR1 axis is necessary for efficient cytotoxic immune response mediated by CD8^+^ T cells (Lei and Takahama, 2012). Also, CCR2 is indispensable for macrophage-dependent inflammatory reactions and regulates monocyte and macrophage recruitment (Weisberg et al., 2006), and has been correlated with delayed AIDS progression in HIV infection (Faure et al., 2000; Arenzana-Seisdedos and Parmentier, 2006). The expression of CX3CR1 appears to induce both adhesion and migration of leukocytes (Imai et al., 1997). Considering the potential roles of these chemokine receptors in innate immunity and adaptive immunity against HIV infection, etc., it’s reasonable to infer that upregulation of these chemokine receptors in MDV-infected birds from the resistant line might suggest a stronger anti-viral response compared to the susceptible line. Meanwhile, the over-expressed interleukin receptor IL1R2 in the resistant chicken spleen indicates the regulation of inflammatory response; researchers have revealed IL1R2’s function as a proinflammatory factor by activating several inflammatory cytokines’ expression (Mar et al., 2013). Also, in the chicken spleen, the upregulated IL4R (ENSGALG00000006313) promotes differentiation of Th2 cells (Fernandez-Botran et al., 1988), and IL21R (ENSGALG00000006318) fosters T cells, B cells, and natural killer cells’ proliferation and differentiation (Parrish-Novak et al., 2000).

Other interesting genes include signal transducer and activator of transcription 1 (STAT1) and leukemia inhibitory factor (LIF). STAT1 was discovered through its involvement in interferon (IFN) signaling and its function in regulating cell growth and differentiation, immune response, antiviral activity, and homeostasis (Ramana et al., 2000). Challenged with chemical carcinogens, mice lacking STAT1 showed more rapid and frequent tumor development (Lee et al., 2000). There was little or no STAT1 expression even after IFN treatment in some tumor cells and tumor-derived cell lines, and STAT1-depleted cells resisted apoptosis in response to TNF or IFN-gamma (Lee et al., 2000; Sun et al., 1998). ChIP-seq experiments using IFN-stimulated HeLa S3 cells indicated the genome-wide binding sites of STAT1 mainly fall into promoters and intronic regions, and suggested the high complexity of STAT1-mediated gene regulatory mechanism (Satoh and Tabunoki, 2013). Leukemia inhibitory factor (LIF) was observed to stimulate proliferation of breast, kidney, and prostate cancer cells (Kellokumpu‐Lehtinen et al., 1996). LIF inhibited Th17 cells differentiation by exerting an opposite effect on STAT3 phosphorylation, which is required for Th17 cell differentiation, in experimental autoimmune encephalomyelitis mice (Cao et al., 2011). When examining STAT1 and LIF expression levels in spleen tissues from the resistant chickens, it is interesting to note that STAT1 was overexpressed (with a logFC of 1.43), and LIF was downregulated (with a logFC −3.33) compared to the non-infected control birds. In resistant chickens, the high level of STAT1 might promote the expression of immune-related genes to fight against MDV while the inhibition of LIF could activate T cell differentiation under the MDV challenge. Since these two genes were only differentially expressed in resistant rather than susceptible chicken lines, their biological functions may contribute to the phenotypic variations induced by MDV infection.

In the three immune organs, we discovered common pathways including cytokine-cytokine receptor interactions and cell adhesion molecules (CAMs) induced by MDV infection. The cytokines and cell adhesion molecules indicate the involvement of innate as well as adaptive inflammatory host defense against MDV infection and immune cell-cell interactions. In fact, most DE genes (as shown in Figure 5) and enriched pathways (Appendix I) identified in our study were tissue-specific. For instance, Herpes simplex infection and Influenza A pathways were mainly enriched in MD-resistant line 6_3_ chicken spleen tissues, while tight junction and focal adhesion were enriched in MD-resistant line 6_3_ chicken bursa tissues. In MD-susceptible line 7_2_ chickens, pathways associated with metabolism, cell cycle, and DNA replication were highly enriched in three organs induced by MDV infection. This observation is in line with previous findings that networks related to cell-mediated immune response were specifically enriched in MD-resistant line 6_3_ chickens (Yu et al., 2011; Mitra et al., 2015).

Although in recent years many studies examining gene expression changes related to MD have revealed the interaction between virus and hosts to some extent, the results from similar experiments vary significantly. Admittedly, the complex nature of Marek’s disease introduces confounding sources of variations, such as virus strains, the genetic background of birds and experimental procedures. Hence, it is arbitrary to compare results from experiments performed under different circumstances simply.

In summary, we have carried out a comprehensive analysis of three immune organs’ transcriptomes in inbred chicken lines, showing differential reactions to MD with a focus focusing on the spleen tissue because all stages of the MDV life cycle occur in the spleen (Baigent and Davison, 2004). We designed and performed a pair-wise experiment based on chicken lines and infection time to control the intrinsic transcriptional fluctuation and take full advantage of the similar genetic background of these inbred lines. This methodology enabled us to characterize genes highly associated with MD resistance and susceptibility and reveal a universal impact of MDV infection on the hosts. Using DAVID and IPA analysis, we observed remarkable distinctions between two lines in the natural state and in response to MDV infection. We discovered enriched networks in metabolism, tissue development, gene expression, and cell signaling. Although our data have provided candidate genes and pathways controlling the different physiological responses between two chicken lines, functional studies are necessary to validate the impacts of those intriguing genes/pathways and elucidate the underlying mechanism associated with MD resistance.

## Data Availability

The original contributions presented in the study are publicly available. This data can be found here: NCBI SRA Repository, accession numbers SRR32499841, SRR32499840, SRR32499829, SRR32499824, SRR32499823, SRR32499822, SRR32499821, SRR32499820, SRR32499819, SRR32499818, SRR32499839, SRR32499838, SRR32499837, SRR32499836, SRR32499835, SRR32499834, SRR32499833, SRR32499832, SRR32499828, SRR32499827, SRR32499826 and SRR32499825.
